# Creating Dementia-Inclusive Communities Using a Geriatric Workforce Enhancement Program Framework

**DOI:** 10.1093/geroni/igab046.1980

**Published:** 2021-12-17

**Authors:** Jennifer Drost, Margaret Sanders

**Affiliations:** 1 Summa Health System, Akron, Ohio, United States; 2 Northeast Ohio Medical University, ROOTSTOWN, Ohio, United States

## Abstract

The Geriatric Workforce Enhancement Program (GWEP) sponsored by HRSA provides an organizing framework around which dementia inclusive community initiatives can be successfully implemented and sustained. The overarching goal of all GWEPs is to improve outcomes for older adults by promoting evidence-based education that spans the continuum of care. This includes integration of academic, clinical, and community-based providers. By their very nature, all GWEPs partner across agencies throughout the state to deliver interprofessional education that will impact people living with dementia and their caregivers at the community, primary care, and acute care levels. Dementia inclusive community initiatives must have this kind of high-level interagency coordination. Our GWEP has successfully implemented Dementia Friends sessions across multiple sectors (Veterans, EMS, clergy, libraries, developmentally disabled, living alone) both in-person and virtually due to COVID-19. This symposium will share the methods to organize at the community level to deliver a unified message community-wide.

